# Managing Menopause: The Evolving Role of Estrogens, Selective Serotonin Reuptake Inhibitors, and Phytoestrogens in Balancing Hormonal Fluctuations

**DOI:** 10.7759/cureus.70440

**Published:** 2024-09-29

**Authors:** Cassande Besong, Sandy Philippeaux, Ansa Bham, Naicha Gustinvil, Abigayle Castine, Giustino Varrassi, Patricia Sutker, Benjamin C Miller, Caroline R Burroughs, Sonja Gennuso, Sahar Shekoohi, Alan D Kaye

**Affiliations:** 1 School of Medicine, Ross University, Miramar, USA; 2 School of Medicine, Ross University, Bridgetown, BRB; 3 School of Medicine, Louisiana State University Health Sciences Center, Shreveport, USA; 4 Pain Medicine, Paolo Procacci Foundation, Rome, ITA; 5 School of Medicine, Louisiana State University Health Sciences Center, New Orleans, USA; 6 Department of Anesthesiology, Louisiana State University Health Sciences Center, Shreveport, USA

**Keywords:** estrogen, menopausal hormone therapy, menopausal nonhormonal therapy, menopause, phytoestrogen, ssris, symptoms

## Abstract

Menopause is characterized by the cessation of menstruation for 12 consecutive months. In this narrative review, we describe the transitional stages of menopause, clinical presentation, pharmacological management of symptoms, and effects on fluctuating hormone levels. To standardize the stages of menopause, the Stages of Reproductive Aging Workshop + 10 (STRAW+10) system was designed with five distinct categories corresponding to symptom presentation and numerical years. Common clinical presentations consist of vasomotor hot flashes, mood changes, decreased libido, osteoporosis, and genitourinary changes, all of which are associated with changes in hormone levels. Because hormones play a major role in the mechanism of menopause, hormone replacement therapy with estrogen, progesterone, or a combination is explored and shown to demonstrate symptom reduction in a large percentage of women. Nonhormonal treatment and alternative therapies are used to treat vasomotor symptoms if contraindications present to hormone therapy or for those who prefer fewer side effects. Thus, many women experience uncomfortable symptoms during menopause, some of which cause significant changes in quality of life. In this regard, understanding the pathophysiology, symptomatology, and current treatment options with side effect profiles allows for continued research and discovery of advanced therapy for treating symptoms to ameliorate discomfort and pain in menopausal women.

## Introduction and background

Menopause is a normal physiological process that marks the permanent cessation of a woman's menses. This process is characterized by the absence of a menstrual cycle for 12 consecutive months. Approximately 95% of women reach menopause between the ages of 45 and 55, with an average onset age of 51. Menopause is related to nonpathologic estrogen deficiency that results from the depletion of oocytes. This creates a hypothalamic-pituitary axis with decreased sensitivity to both positive and negative effects of estrogen, leading to no luteinizing hormone (LH) surge or ovulation and anovulatory menstrual cycle patterns [1]. Moreover, LH and follicle-stimulating hormone (FSH) go uninhibited and remain at high levels years after the onset of menopause [2]. This narrative review, therefore, provides an in-depth analysis of menopause, including its pathophysiology, transitional stages, associated symptoms and treatment options, and their effect on balancing fluctuations in hormone levels. Understanding these aspects is critical for effective management and improvement in quality of life for all women experiencing menopause. With this review, we discuss effective treatment options currently available to women seeking symptom relief and provide patients with information to become better informed and thus make better educated decisions regarding their health.

## Review

Transitional stages of menopause

The menopausal transition is characterized by hormonal changes and clinical symptoms [3]. To standardize the different transitional stages of menopause, the Stages of Reproductive Aging Workshop + 10 (STRAW+10) was formulated [1]. STRAW is from other studies of the menopausal transition and has clinically applicable aspects based on menstrual bleeding patterns. The stages from STRAW that have a numerical delineation along with the symptoms include the following: late reproductive stage (−3), the early menopausal transition (−2), the late menopausal transition (−1), the final menstrual period (FMP; 0), the early postmenopause (+1) [3].

The earliest stage (−3) is the late reproductive years [3]. The onset of this stage may occur after age 25 since the antimullerian hormone peaks at this age and then continuously declines [3]. The number of follicles for ovulation is mostly preserved at this stage; thus, FSH is normal and sometimes increased [1]. Menopause is the next major stage, divided into the early and late transition stages. For the average woman, the early transition stage (−2) is at age 47, while the late transition stage (−1) is at age 49, and the FMP (stage 0) is at age 51; however, these ages may vary [1]. The early menopausal transition is characterized by a difference of seven or more days in a woman's menstrual cycle length. A skipped menstrual period is the first sign, along with irregular bleeding patterns, such as changes in bleeding frequency and duration, which usually alert the woman to seek medical care [1]. The irregular bleeding patterns tend to be associated with anovulation. At the late menopausal transition stage (−1), the menstrual cycle becomes even more irregular. It is defined as when a woman experiences a period of amenorrhea at or greater than 60 days, which increases in duration and lasts approximately 1-3 years [1]. At this stage, FSH is more consistently elevated, while circulating estrogen is low in anovulatory women. In addition, long periods of amenorrhea cause an increase in the prevalence of common menopausal symptoms. The final stage, the early postmenopausal stage, is defined as when 12 consecutive months have passed without a menstrual cycle.

Clinical presentation of menopause

Menopause is linked to a variety of symptoms caused by modifications in hormone levels, particularly a decrease in estrogen. Symptoms of the menopausal transition affect more than 80% of women [3]. As previously mentioned above, the transition period is associated with an enhancement in common menopausal symptoms such as vasomotor symptoms (VMS; hot flashes), genitourinary symptoms, mood changes, decreased libido, and osteoporosis [3].

VMS affect approximately 80% of women worldwide [4]. VMS can cause disturbed sleep, fatigue, and depressed mood. The prevalence of VMS may vary by race/ethnicity. African American and Hispanic women may experience a longer duration of symptoms as opposed to non-Hispanic White, Chinese, and Japanese women, who reported a significantly lower duration [4]. These prolonged symptoms may significantly negatively affect overall health and perceived well-being. Women who experience frequent symptoms, defined as more than six days in the previous two weeks, can also experience comorbidities such as depression, anxiety, and difficulty sleeping, all of which can have an overall negative effect on their quality of life [4]. VMS are also associated with greater cardiovascular risks. This may be due to loss of endothelial function during menopause, possibly related to loss of estrogen [1]. Among the various interventions available, hormone replacement therapy (HRT) is the most effective treatment for management [1].

Genitourinary symptoms affect about 50% of menopausal women and remain significantly underreported [1,3]. The connective tissue dehydration due to lack of secretions then causes narrowing of the vagina along with atrophy of the vulva. Thus, increased vaginal pH and vaginal atrophy can increase the risk of urinary tract infections (UTIs) [1]. An increase in UTIs is likely due to a decrease in lactobacilli, normal vaginal flora responsible for protection against foreign bacteria [5]. Consistent and timely doses of vaginal estrogen in postmenopausal women with a diagnosis of recurrent UTIs have been shown to prevent UTI occurrences in this population [6]. Vaginal estrogen replacement in low doses is absorbed very little by the systemic circulation. Thus, it is unlikely to cause gross endometrial hyperplasia and other symptoms such as stroke, thromboembolism, or cardiovascular risks [5,7]. Because of its anatomical location and minimal absorption, vaginal estrogen can bypass the liver and, thus, first-pass metabolism, likely lending to a favorable decrease in the side effect profile compared to systemic estrogen [7].

The major mood symptom related to menopause is depression. Women with no prior history of depression have an increased risk of acquiring a new onset of depression or anxiety during menopause. Studies have shown an enhanced risk of depressive symptoms in peri- or postmenopausal women in comparison with premenopausal cases from the Study of Women's Health Across the Nation (SWAN). The adverse side effect of depression is developing major depressive disorder, which can be life-threatening and was only observed in 3% of women at the SWAN baseline assessment. HRT may improve mood without substantially increasing VMS in perimenopausal women [1].

During the transition phase of menopause, approximately 10% of women experienced decreased sexual drive [1]. The decline in estrogen can also cause sexual dysfunction, such as dyspareunia, reduced libido, arousal difficulties, and difficulties achieving orgasm [1,8]. Testosterone levels also decrease with age; however, there is no significant relationship between sexual activity and reduced testosterone levels in menopause. Starting testosterone replacement shows minimal to no change in symptoms [3]. Other causes of decreased libido include emotional fluctuations, vaginal atrophy, and chronic diseases [3]. Currently, there are no FDA-approved drugs that treat low libido in postmenopausal women.

One of the final major symptoms of menopause involves osteoporosis. Osteoporosis is characterized by either a fracture or low bone mineral density. The cause of osteoporosis is a lack of ovarian estrogen after menopause, which leads to a significant loss of bone strength [9]. Osteoporotic fractures affect half of women after age 50. The rates of bone loss significantly increased from a year preceding the FMP and remained elevated for up to three years at a rate of 5% per year, then slowed again to the loss rate before menopause [1]. Also, during the onset of the menopausal period, women lose approximately 25%-30% of their trabecular bone and 10%-15% of their cortical bone reserves. The most common concern in osteoporosis is fractures that prevalently occur in the hip joint, a hazardous anatomical site in older ages [10]. Other commonly fractured sites include the wrist and vertebrae of the spine, which contain spongy bone more susceptible to fractures when weakened by decreased bone mineral density [11]. Nonpharmacologic treatment (calcium and vitamin D supplements), smoking cessation, adequate exercise, and fall prevention [12] are recommended. Adequate exercise, specifically weight-bearing and of long duration, has been shown to maintain or, in some cases, increase bone mineral density in postmenopausal women [13]. Pharmacologic treatments include bisphosphonates, parathyroid hormone, estrogen, and calcitonin [12]. Interestingly, the use of nonpharmacologic therapy, such as physical exercise with medication, confers a critical role in the treatment of postmenopausal osteoporosis compared to medication alone [11].

Pharmacological management for menopause

Hormonal Treatment for VMS

The most effective treatment for hot flashes is estrogen replacement therapy (HRT). Estrogen replacement therapy can reduce hot flash symptoms by up to 75% [14]. Unfortunately, there are many side effects, complications, and contraindications when it comes to HRT. Some of the complications are increased risk of venous thrombotic disease, breast cancer, stroke, and coronary artery disease. Although the use of estrogen therapy brings along many risks, women with severe VMS have no other treatment available; because of that, a low dose of estrogen is used [14]. The utilization of estrogen therapy in breast cancer survivors remains a controversial topic due to its vast side effect profile.

Another pharmacological agent that is used in conjunction with estrogen replacement therapy is progesterone. Progesterone is highly recommended in menopausal women with an intact uterus to counteract the effect of estrogen and decrease the risk of endometrial hyperplasia and carcinoma [5,15]. The protective effect of progesterone on the endometrium is dependent on dose and duration [5]. Furthermore, much research has proven that progesterone can be used as a monotherapy treatment for VMS in menopausal women. Examples of progesterone that were tested in the trial were megestrol acetate and medroxyprogesterone acetate. Both were found to have an 85%-90% reduction of hot flash symptoms compared to the placebo [16]. However, despite the great benefits, the use of progesterone comes with similar side effects and risks as administering estrogen. Those risks can vary from venous thrombotic disease, breast cancer, stroke, and coronary artery disease. Because of the risks associated with use, these hormone replacement therapies are contraindicated in women with liver disease, abnormal uterine bleeding, and thromboembolic disease.

Other risks associated with synthetic progesterone use, also referred to as progestin, include water retention and weight gain related to an additional affinity for mineralocorticoid and glucocorticoid receptors in addition to progesterone receptor activation. Dydrogesterone is a unique synthetic progesterone compound because it does not result in a significant side effect profile, thus remaining more neutral than other synthetic progestins [5]. This neutrality with retained efficacy is essential for menopausal women experiencing symptoms to consider when deciding on an HRT to begin.

Nonhormonal Treatment for VMS

Related to the numerous side effects of HRT, many patients choose to treat their VMS with nonhormonal treatment options. Nonhormonal treatments include antidepressants such as selective serotonin reuptake inhibitors (SSRIs), serotonin and norepinephrine reuptake inhibitors (SNRIs), gabapentin, and clonidine [17,18].

Paroxetine was the first SSRI used to treat VMS and is currently one of only two FDA-approved nonhormonal prescription therapies for this indication: paroxetine mesylate 7.5 mg daily [19]. Paroxetine has shown strong results in clinical trials, proving effective in significantly reducing VMS severity and frequency. More specifically, studies have shown paroxetine to be efficacious in significantly reducing hot flash symptoms, as well as shown promising improvements in patients' sleep quality, anxiety, and depression, without weight gain or adverse effects on libido [20,21]. Prior to starting paroxetine treatment, patients should review their current medication list with their provider. Paroxetine is a strong CYP2D6 inhibitor and can alter concentrations of other drugs metabolized by CYP2D6. Absolute contraindications for paroxetine use include concurrent use of monoamine oxidase inhibitors, thioridazine, and pimozide [22]. Additionally, precautions should be taken with concomitant tricyclic antidepressants (TCA) or tamoxifen use. Paroxetine inhibits the metabolism of TCA, leading to possible TCA toxicity, and blocks the activation of tamoxifen, which requires CYP2D6, reducing the amount of active drug that is released.

Other nonhormonal treatment options for VMS are gabapentin and clonidine. Gabapentin, a drug commonly prescribed to treat neuropathic pain, has proven to be effective in decreasing the frequency of VMS in postmenopausal women [20]. Clonidine, an alpha-2-adrenergic receptor agonist commonly used to treat insomnia, attention-deficit/hyperactivity disorder, post-traumatic stress disorder, and anxiety, can also be used to decrease the severity and frequency of hot flashes in postmenopausal women [23]. One theory for this reduction in hot flashes by using clonidine is that it reduces the vascular reactivity in peripheral vessels without causing significant changes in blood pressure when administered at low doses [23,24]. However, clonidine is limited in clinical use due to its modest effect on symptom improvement and side effects, e.g., weight gain, blurred vision, constipation, and orthostatic hypotension [19,25].

Alternative Therapies for VMS

All pharmacological agents come with many different side effects and contraindications. Because of this, patients may choose alternative therapies with little or no side effects. Many studies have shown that postmenopausal women from Asia present with less VMS compared to women in the United States of America [26]. This is due to compounds called phytoestrogens. Phytoestrogens are naturally occurring compounds with estrogenic and antiestrogenic properties and can be found in many foods consumed in Asia, such as soybeans and soy-based dishes [26]. Possessing a chemical structure similar to estradiol, isoflavones and lignans are the two major classes of phytoestrogens with phenolic structure and act functionally similar to estradiol. Isoflavones are abundant in soybeans and exhibit the most potent estrogen-like activity among all phytoestrogens, whereas lignans, found in fruits, vegetables, legumes, flaxseed, and whole grains, are less potent but more diverse [27,28]. Phytoestrogen activity, whether estrogenic or antiestrogenic, depends on the circulating level of physiologic estrogen present. A low level of physiologic estrogen makes phytoestrogen more estrogenic, whereas a high level of physiologic estrogen tends to cause phytoestrogen to act as an antiestrogenic compound [27].

Research was undertaken to assess the effectiveness and tolerability of phytoestrogens in menopausal women, as VMS symptoms during menopause are less prominent in women from Asia, where a diet rich in phytoestrogens is prevalent. Inconclusive results were reported regarding the use of phytoestrogens to treat VMS due to variability in study designs, such as the type of consumed phytoestrogen, dosage of phytoestrogen, dietary consumption of phytoestrogens, and lack of control groups. A randomized controlled trial to analyze the efficacy and tolerability of phytoestrogens in the treatment of VMS reported phytoestrogens “significantly reduce the frequency of hot flushes compared with the placebo” with “no difference in the occurrence of side effects between patients who received phytoestrogens and those who received placebo” (Table 1) [27].

**Table 1:** Summary of agents used in menopause, mechanism of actions, clinical indications, and side effects VMS: vasomotor symptoms; SSRIs: selective serotonin reuptake inhibitors; GI: gastrointestinal; PTSD: post-traumatic stress disorder; OCD: obsessive compulsive disorder; EPS: extrapyramidal symptoms Table credits: The table was created by the authors Cassande Besong, Ansa Bham, and Sandy Philippeaux

Drug/hormone name	Mechanism of actions	Clinical indications	Side effects
Estrogen	In the reproductive system, it acts by altering the transcription of genes that promote development, regulation, and maintenance of the uterus and vagina	VMS (menopause), hormone therapy, and osteoporosis	Headache, bloating, fatigue, GI upset, and increase risk of breast, ovarian, and endometrial cancer
SSRIs	Inhibition of the reuptake of serotonin at the serotonin transporter SERT located at the presynaptic axon terminal, thus increasing serotonin activity	Depression, hot flashes (menopause), PTSD, panic disorder, and OCD	Serotonin syndrome, EPS, QT prolongation, suicidal idealizations, dizziness, headaches, dry mouth, loss of libido, and feeling agitated
Gabapentin	Blocks presynaptic voltage-gated calcium channels resulting in inhibition neurotransmitter release and neuronal signaling	Neuropathy, VMS (menopause), restless leg syndrome, restless tremors, and seizures	Dizziness, drowsiness, fatigue, headache, and abdominal discomfort.
Phytoestrogens	Plant-based substance that mimics circulating estrogen by weakly activating estrogen receptors in mammals	VMS (menopause)	Allergic reactions, abdominal pain, muscle pain, and drowsiness

## Conclusions

Menopause is characterized as the permanent cessation of menses for at least 12 consecutive months, characterized by a period of hormonal fluctuations and VMS. While the average age of menopausal onset is 51, the transition can commence as early as a woman's 30s and last for over a decade. Premature menopause is different from primary ovarian insufficiency, which occurs when a woman's ovaries stop working normally before she is 40. Menopause constitutes a significant transition in a woman's life and brings about substantial changes, often manifesting as uncomfortable and distressing symptoms. VMS, commonly referred to as hot flashes and night sweats, are common during menopause and remain undertreated in the aging female population. A comprehensive understanding of pathophysiology, clinical manifestations, and available treatment options is critical for effective management. It is also vital that clinicians recognize the diverse needs of individuals experiencing menopause and develop personalized treatment plans accordingly. Hormone therapy remains the first-line recommended treatment to mitigate VMS in healthy menopausal women. However, not all women are candidates for hormone therapy due to either contraindications or personal preferences and can instead select from a variety of effective nonhormonal treatment options, including SSRIs, SNRIs, and gabapentin. The purpose of this review was to discuss our current understanding of menopause and available treatment options and highlight the need for further research and advancements in drug development. Future efforts should be directed toward patient education, early intervention, and long-term management of menopause. Providers must begin implementing a more personalized approach to patient care to address the unique menopausal needs of each patient. Doing so will lead to more effective management strategies and treatments for menopause. Women must be vocal about their menopausal concerns and needs, and clinicians, in return, must be open and available to address each of these needs.
